# A randomized phase II trial of hepatic arterial infusion of oxaliplatin plus raltitrexed versus oxaliplatin plus 5-fluorouracil for unresectable colorectal cancer liver metastases

**DOI:** 10.3389/fonc.2022.913017

**Published:** 2022-09-21

**Authors:** Ai-Wei Feng, Jian-Hai Guo, Song Gao, Fu-Xin Kou, Shao-Xing Liu, Peng Liu, Hui Chen, Xiao-Dong Wang, Hai-Feng Xu, Guang Cao, Xu Zhu

**Affiliations:** Department of Interventional Therapy, Key Laboratory of Carcinogenesis and Translational Research (Ministry of Education/Beijing), Peking University Cancer Hospital and Institute, Beijing, China

**Keywords:** hepatic arterial infusion, colorectal cancer, liver metastases, oxaliplatin, raltitrexed, 5-fluorouracil

## Abstract

**Background:**

The purpose was to compare the efficacy and safety of hepatic arterial infusion (HAI) of oxaliplatin plus raltitrexed (TOMOX) to those of oxaliplatin plus 5-fluorouracil (FOLFOX) for unresectable colorectal cancer liver metastases (CRCLM).

**Methods:**

Patients with unresectable CRCLM were randomly assigned to receive HAI of TOMOX or FOLFOX. The primary end points were progression-free survival (PFS) measured from the date of randomisation until the date of disease progression and objective response rate (ORR). The secondary end points were overall survival (OS) measured from the date of randomisation until the date of death from any cause, disease control rate (DCR), and adverse events.

**Results:**

113 patients were randomly assigned. With a median follow-up of 39.5 months, the PFS was 5.8 months [95% CI, 4.838–6.762]) and 4.6 months [95% CI, 3.419–5.781; P = 0.840], and the median OS was 17.6 months [95% CI, 13.828–21.372] and 13.1 months [95% CI, 11.215–14.985; P = 0.178] for the FOLFOX and TOMOX arm, respectively. The ORR were 26.1% vs 22.4% and DCR were 80.4% vs 71.4% in the FOLFOX and TOMOX arms. The most common severe adverse event was elevation of liver enzymes and pain, which did not differ in the two arms.

**Conclusion:**

HAI chemotherapy was effective for unresectable CRCLM. HAI of FOLFOX has similar efficacy to TOMOX, and HAI of TOMOX had shorter arterial infusion time.

**Clinical Trial Registration:**

https://clinicaltrials.gov/, identifier NCT02557490.

## Background

Colorectal cancer is the third most commonly diagnosed cancer and the second leading cause of cancer death all over the world (1). Approximately 30% of all patients with colorectal cancer develop liver metastases, liver lesions account for at least two-thirds mortality (2). Given that liver resection is associated with improved prognosis, systemic chemotherapies combining with targeted therapies (anti-vascular endothelial growth factor (anti-VEGF) or anti-epidermal growth factor receptor (anti-EGFR) therapy), and HAI chemotherapy has been focused on improving the potential for resection of liver metastases considered unresectable (3–6). The blood supply of liver parenchyma is mainly from portal vein, but the blood supply of liver tumour is mainly from hepatic artery (7). For patients with liver-only or liver-dominant metastases, HAI chemotherapy has evolved as an attractive local therapeutic option because of low systemic toxicity and high local control rates, even when all standard systemic therapy has been used (8–11).

Floxuridine has been widely used for HAI because of the high first-pass hepatic extraction and limited systemic toxic effects noted for the drug, regardless of whether HAI is used alone or in combination with systemic chemotherapy (12–14). The most common adverse events of HAI of floxuridine are biliary toxicity and biliary sclerosis, which are non-interventional and permanent. Several clinical studies showed that the incidence of these adverse events increased when the treatment also included systemic bevacizumab (15–17). Oxaliplatin, irinotecan, 5-fluorouracil, and raltitrexed are the main chemotherapeutic drugs for colorectal cancer; HAI is gradually being used for these drugs, especially in Asia and Europe. Patients who underwent postoperative adjuvant HAI of oxaliplatin combined with systemic chemotherapy showed significantly better 3-year disease-free survival after radical resection of colorectal cancer liver metastases (CRCLM) than patients who underwent adjuvant systemic chemotherapy alone (18). HAI along with doublet or triplet chemotherapy is still extremely effective, even for cases of unresectable CRCLM that have not responded to previous chemotherapy (6, 19).

HAI of FOLFOX has been found to be a feasible treatment option for unresectable CRCLM. 5-fluorouracil is administered intra-arterially for approximately 44 hours every cycle and oxaliplatin is for 4 hours. However, prolonged bed rest increases the incidence of thromboembolic events in some high-risk patients. HAI of TOMOX can help avoid thromboembolic events because raltitrexed requires a short pumping time for only 1 hour. We had previously conducted a retrospective study at our centre, wherein a head-to-head analysis comparing HAI of FOLFOX with TOMOX for unresectable CRCLM had been performed; PFS and OS were found to be similar in both arms (20). Therefore, we expanded on that analysis in the current prospective randomised controlled trial, which aimed to further compare the efficacy and safety of HAI of FOLFOX with TOMOX for unresectable CRCLM.

## Patients and methods

### Ethics approval

This study was initiated by Beijing Cancer Hospital, China, and registered at ClinicalTrials.gov (identification number, NCT02557490). Informed consent was obtained from the study participants, and the study protocol was approved by the local ethics committee.

### Patients

The inclusion criteria were as follows: age ≥ 18 years; histologically confirmed colorectal adenocarcinoma with unresectable liver metastases occupying less than 70% of the liver parenchyma; Eastern Cooperative Oncology Group (ECOG) performance score < 2; life expectancy > 12 weeks; haemoglobin level > 90 g/L; absolute neutrophil count > 1.5×10^9^/L; thrombocyte > 80×10^9^/L; liver enzyme (including alanine amino transferase and aspartic acid amino transferase) level < five times of the upper limit of the normal range; total bilirubin level < three times of the upper limit of the normal range; serum creatinine level < 1.5 times of the upper limit of the normal range; and prothrombin time < 1.5 times of the upper limit of the normal range; refractory or intolerant to systemic treatment; or unsuitable for systemic treatment. Patients who had extrahepatic metastases were included at the investigators’ discretion, provided that the dominant lesion was hepatic.

The main exclusion criteria were as follows: (a) brain metastases, (b) previous trans-arterial chemoembolization (TACE), and (c) other malignancy (within 3 years before study entry).

### HAI procedure

For the HAI procedure, a coaxial catheter (Renegade Hi Flo, Boston Scientific, Boston, MA, United States/Stride ASAHI INTECC, Seto, Japan) was inserted through the femoral artery using Seldinger’s technique. Based on the tumour location, a microcatheter was placed in the proper hepatic artery or the right or left hepatic arterial branch under arteriography guidance. The peripheral region of the microcatheter that was exposed outside the body was connected with an arterial chemotherapeutic pump. Medication infusion was initiated immediately after catheter insertion. The microcatheter was removed at the end of every treatment cycle.

### Medication protocol

The FOLFOX arm consisted of oxaliplatin (85 mg/m^2^ *via* 4-h infusion) and 5-Fluorouracil (2000 mg/m^2^ *via* 44-h infusion) administered *via* HAI and leucovorin (200 mg/m^2^ *via* 2-h infusion initiated at the beginning of the 5-Fluorouracil infusion) administered *via* intravenous infusion. The TOMOX arm consisted of oxaliplatin (85 mg/m^2^ *via* 4-h infusion) and raltitrexed (3 mg/m^2^ *via* 1-h infusion) administered *via* HAI.

Compared to systemic chemotherapy, HAI can increase the local blood concentration. The incidence of liver injury can be reduced and the treatment tolerance is better improved with the prolonged interval. HAI was regularly performed every 4 weeks, until disease progression, treatment intolerance, or death occurred.

### Objectives and assessment

The primary end points were progression-free survival (PFS) defined as the date of randomisation until the date of disease progression and ORR defined as the proportion of patients achieving complete response (CR) or partial response (PR). The secondary end points were overall survival (OS) defined as the date of randomisation until the date of death from any cause, DCR and adverse events. DCR was defined as the proportion of patients achieving CR, PR, or stable disease (SD). Tumour response to treatment was evaluated by imaging analysis according to Response Evaluation Criteria in Solid Tumour (RECIST) version 1.1. Adverse events were categorized on the basis of Common Terminology Criteria for Adverse Events (CTCAE) 5.0.

Pre-treatment evaluation included laboratory tests, chest computed tomography (CT), abdominal CT or magnetic resonance imaging (MRI). Laboratory tests were performed every week during the treatment. Imaging analyses for all lesions (intrahepatic and extrahepatic lesions) were performed for every cycle. Additional imaging analyses were performed to detect potential metastases if clinical symptoms appeared.

### Statistical analysis

The assumptions used for size calculation are following: the median PFS in the FOLFOX arm was about 7 months while the median PFS in the TOMOX arm was about 4 months, bilateral α=0.05, power = 80%. The enrollment period was 36 months, the minimal follow up period was 12 months, the total study period was 48 months. Loss to follow-up rate was set as 5%. Using a treatment allocation of 1:1(FOLFOX to TOMOX), total 120 patient in this study and 60 patients per arm were necessary according to the calculation with NCSS-PASS 11 (21, 22).

The χ^2^ test or Fisher’s exact test was used to analyse differences between categorical variables. Survival time was calculated using the Kaplan–Meier method and compared with the Cox regression model (with hazard ratios [HRs] and 95% confidence intervals [CIs] indicated). The significance of differences in survival was calculated using the log-rank test. Potential prognostic variables were included in the univariate Cox regression model. All statistical tests were two-sided and P-values < 0.05 were considered significant. All statistical analyses were performed using the SPSS software (version 25; IBM SPSS Statistics, Armonk, NY, United States).

## Results

### Baseline demographic and clinical characteristics

**Figure 1:**
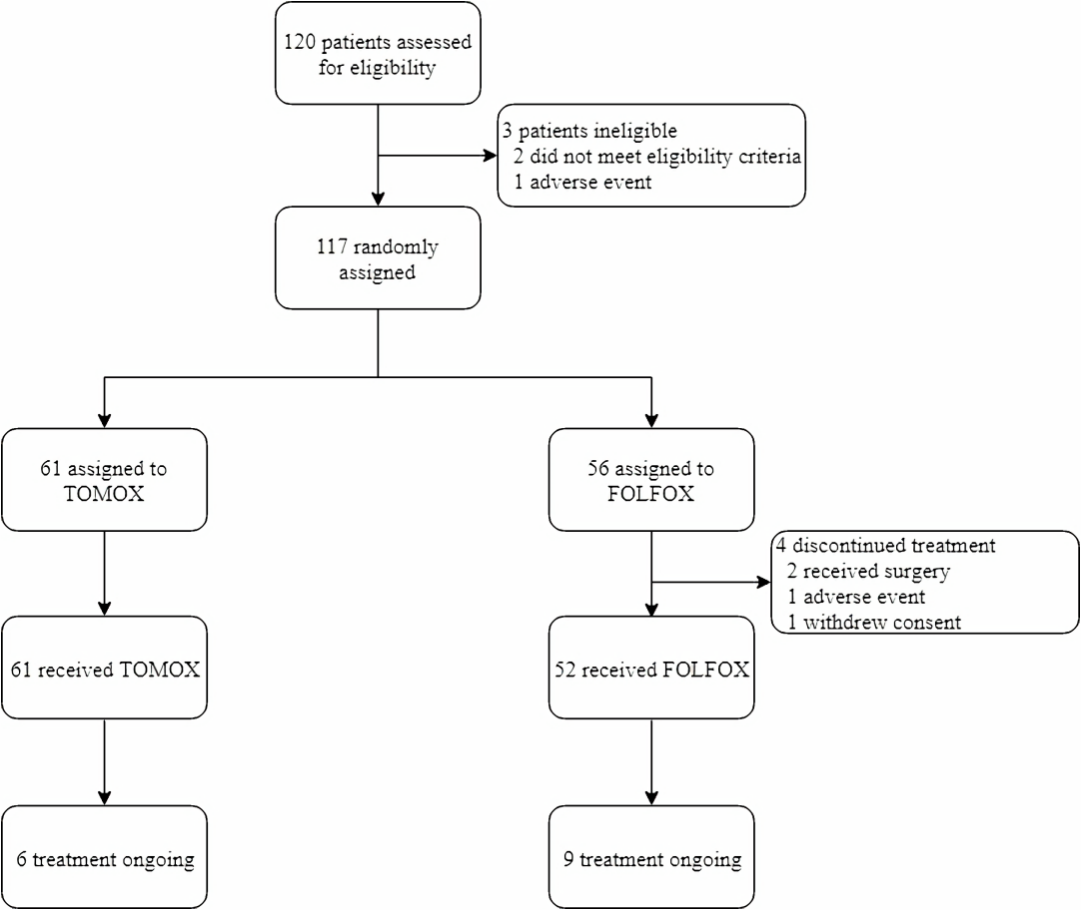
Trial Profile.

From January 2015 to August 2019, 120 patients were screened, of whom 117 patients were randomly assigned to the TOMOX and FOLFOX cohorts (TOMOX arm, n = 61; FOLFOX arm, n = 56). In the FOLFOX arm, four patients were excluded: one patient was allergic to oxaliplatin, two patients underwent surgery after the first treatment cycle without tumour assessment, and one patient withdrew informed consent (**Figure 1**). In the TOMOX and FOLFOX arms, more than 90% of the patients had received oxaliplatin and fluorouracil-based chemotherapy, 78 patients were refractory to systemic therapy, 34 patients were intolerant to systemic therapy. Five untreated patients enrolled in this trial did not have extrahepatic lesion and the hepatic tumour burden was really heavy. After carefully evaluation by multi-disciplinary treatment, HAI was recommended to treat the hepatic metastases without systemic therapy. The patients had also received targeted biologic therapy before HAI, including anti-VEGF therapy (bevacizumab: 26% vs 33%), anti-EGFR therapy (cetuximab: 20% vs 15%), or a combination of both (5% vs 4%) (**Table 1**). There were 58 patients combined with extrahepatic metastases, including lung metastases (34.5%), lymphatic metastases (16.8%), bone metastases (4.4%) and peritoneal metastases (3.5%). All these patients were strictly evaluated by two senior attending physicians independently, and hepatic metastases were the dominant lesions.

**Table 1:** Patient demographics and baseline characteristics.

n (%)	TOMOX n = 61	FOLFOX n = 52
**Age at diagnosis**		
≥65 years <65 years Median age (years [IQR]) **Sex** Male Female **Primary tumour site** Left hemicolon Right hemicolon Unknown **Genetic condition** KRAS mutation KRAS wild type Unknown **Histology** Poorly differentiated adenocarcinoma Well and moderately differentiated adenocarcinoma Unknown **Mean CEA** **Liver metastasis** Synchronous Metachronous **Mean size of the biggest liver metastasis** **No. of metastatic lesions** ≥3 <3 **Primary tumour** Resection No resection Unknown **Extrahepatic metastasis** Present Absent **Systemic therapy before HAI** Untreated First-line Second-line Third-line and above **Previous chemotherapy agents before HAI** Oxaliplatin Irinotecan Fluorouracil **Previous anti-VEGF or anti-EGFR treatment or both before HAI** Bevacizumab Cetuximab Both None	12 (19.7%) 49 (80.3%) 58 (31-79) 41 (67.2%) 20 (32.8%) 41 (67.2%) 18 (29.5%) 2 (3.3%) 13 (21.3%) 14 (23.0%) 34(55.7%) 9 (14.8%) 40 (65.6%) 12 (19.7%) 616.42ng/ml 50 (82.0%) 11 (18.0%) 51.2mm 49 (80.3%) 12 (19.7%) 42 (68.9%) 14 (23.0%) 5 (8.2%) 31 (50.8%) 30 (49.2%) 4 (6.6%) 27 (44.3%) 22 (36.1%) 8 (13.1%) 55 (90%) 33 (54%) 57 (93%) 16 (26%) 12 (20%) 3 (5%) 30 (49%)	11(21.2%) 41(78.8%) 58(34-83) 34(65.4%) 18 (34.6%) 41(78.8%) 9 (17.3%) 2 (3.8%) 16 (30.8%) 11 (21.2%) 25 (48.1%) 8 (15.4%) 33 (63.5%) 11 (21.2%) 604.76ng/ml 47 (90.4%) 5 (9.6%) 48.7mm 42 (80.8%) 10 (19.2%) 33 (63.5%) 15 (28.8%) 4 (7.7%) 27 (51.9%) 25 (48.1%) 1 (1.9%) 20 (38.5%) 19 (36.5%) 12 (23.1%) 48 (92%) 36 (69%) 51 (98%) 17 (33%) 8 (15%) 2 (4%) 25 (48%)

VEGF, vascular endothelial growth factor; EGFR, epidermal growth factor receptor.

### Efficacy

**Figure 2:**
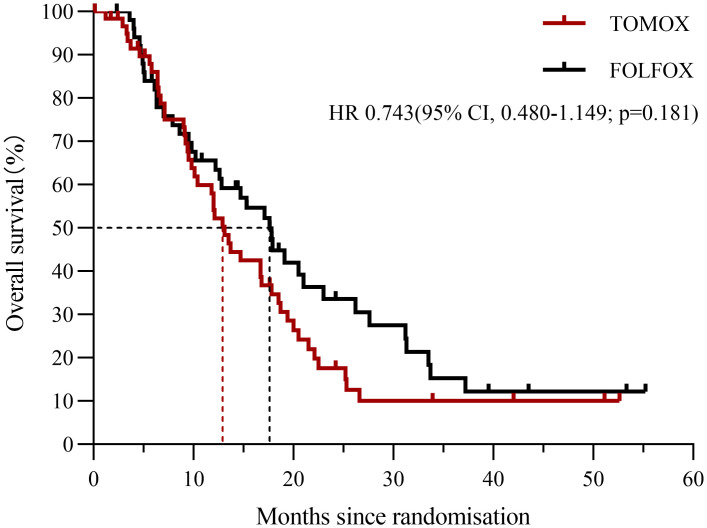
OS since randomization for patients receiving HAI of FOLFOX or TOMOX.

**Figure 3:**
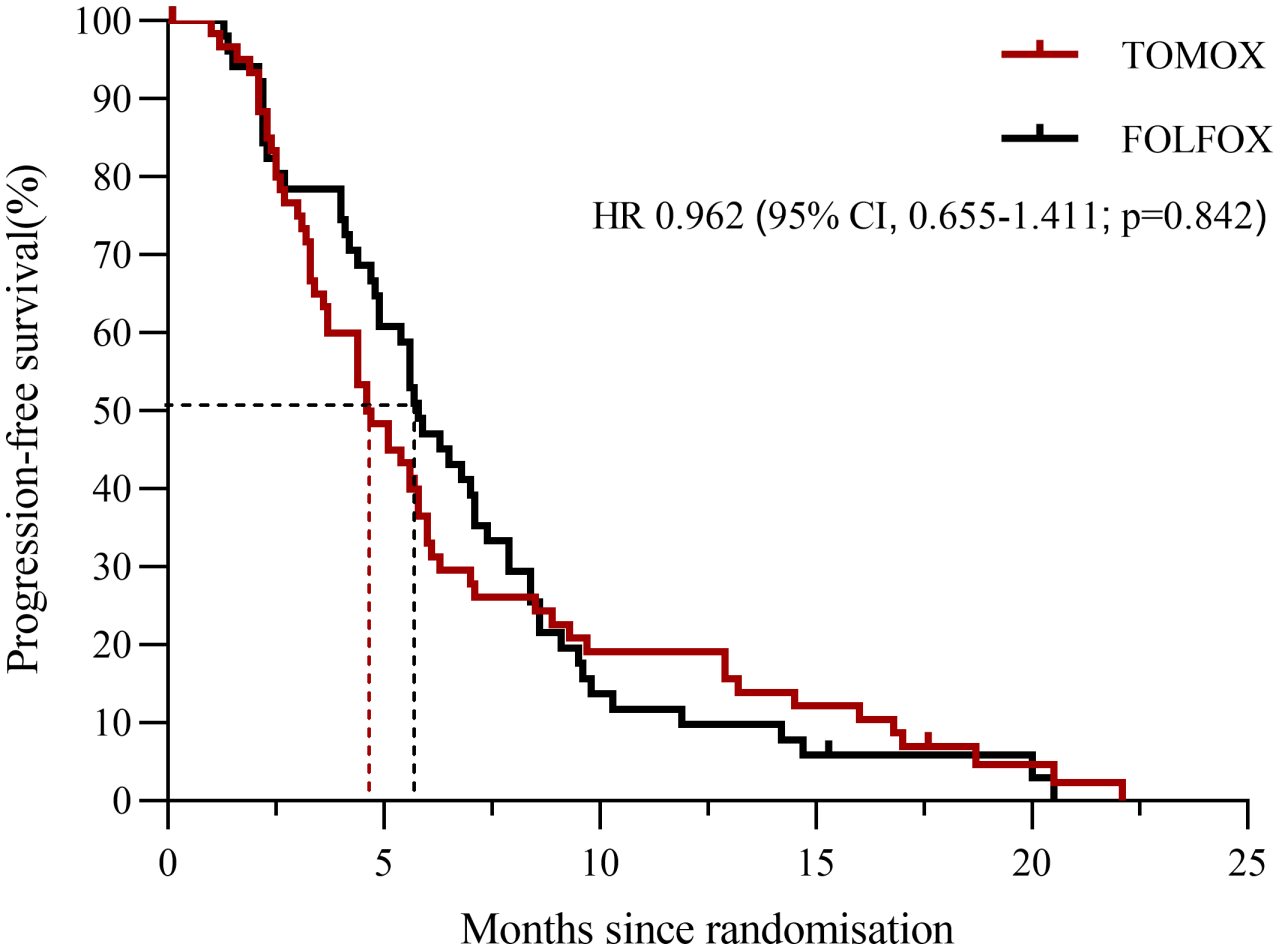
PFS since randomization for patients receiving HAI of FOLFOX or TOMOX.

The final analysis included 113 patients (TOMOX arm, n = 61; FOLFOX arm, n = 52). The cut-off date for follow-up was May 16, 2020 (median follow-up duration, 39.5 months), at which time 83 deaths had occurred. The mean HAI treatment cycles were 3.0 and 2.7 in the TOMOX and FOLFOX arms, respectively. The median OS was 17.6 months [95% CI, 13.828–21.372] in the FOLFOX arm and 13.1 months [95% CI, 11.215–14.985; P = 0.178] in the TOMOX arm (**Figure 2**). The HR for OS was 0.743 for FOLFOX versus TOMOX (95% CI, 0.480–1.149; P = 0.181). The median PFS was 5.8 months [95% CI, 4.838–6.762] in the FOLFOX arm and 4.6 months [95% CI, 3.419–5.781; P = 0.840] in the TOMOX arm (**Figure 3**). The HR for PFS was 0.962 for FOLFOX versus TOMOX (95% CI, 0.655–1.411; P = 0.842). For the patients received HAI as third-line and above therapy, the median PFS was 5.9 months [95% CI, 4.826–6.974] in the FOLFOX arm and 4.4 months [95% CI, 2.793–6.007; P = 0.969] in the TOMOX arm, the median OS was 17.8 months [95% CI, 6.129–29.471] in the FOLFOX arm and 12.9 months [95% CI, 10.617–15.183; P = 0.091] in the TOMOX arm. Two patients in the FOLFOX arm and one patient in the TOMOX arm achieved CR. The ORR was 26.1% in FOLFOX arm and 22.4% in TOMOX arm. The DCR was 80.4% and 71.4% in the FOLFOX and TOMOX arm, respectively (**Table 2**). 1 patient received radical resection of liver metastases. 2 patients who achieved CR received targeted therapy as maintenance therapy without radical resection. 6 patients received radical microwave ablation. 1patient received radical radiotherapy. The other 13 patients who achieved PR did not receive local therapy because of advanced age, unresectable extrahepatic metastases, the remaining liver volume cannot be resected after previous partial hepatectomy and heavy tumour burden cannot be radical resection.

**Table 2:** Response rates to HAI of TOMOX or FOLFOX.

Best response, n (%)	TOMOX n=61	FOLFOX n=52	P-value^a^
CR PR SD PD Unknown	1 (1.6) 10 (16.4) 24 (39.3) 14 (23.0) 12 (19.7)	2 (3.8) 10 (19.2) 25 (48.1) 9 (17.3) 6 (11.5)	0.621
ORR	11 (22.4)	12 (26.1)	
DCR	35 (71.4)	37 (80.4)	

a P-value calculated using a Fisher exact test.

CR, complete response; PR, partial response; SD, stable disease; PD, progressive disease; ORR, objective response rate; DCR, disease control rate.

Response to HAI was an independent positive prognostic factor for both PFS and OS according to Cox univariate analysis (**Table 3**). The primary tumour site, primary tumour resection, and histological features were prognostic factors for OS. However, age, sex, synchronous or metachronous liver metastasis, extrahepatic metastasis, KRAS gene status, and systemic therapy before HAI did not show significant correlation with prognosis.

**Table 3:** Cox univariate analyses of prognostic factors for survival.

Prognostic factor (n)	PFS			OS		
	HR	95% CI	P-value	HR	95% CI	P-value
**Age at diagnosis, years**						
<65 (90) ≥65 (23) **Sex** Male (75) Female (38) **Primary tumour site** Right hemicolon (27) Left hemicolon (82) **Liver metastasis** Synchronous (97) Metachronous (16) **Extrahepatic metastasis** Present (58) Absent (55) **Primary tumour** No resection (29) Resection (75) **Genetic condition** KRAS mutation (29) KRAS wild type (25) **Histology** Poorly differentiated (17) Well and moderately differentiated (73) **Systemic therapy before HAI** ≥Third-line (20) Second line (41) First-line (47) **Response to HAI** CR (3) PR (20) SD (49) PD (23)	1.494 1 0.908 1 1.119 1 0.917 1 1.193 1 1.206 1 0.833 1 1.421 1 0.757 0.942 1 0.034 0.052 0.081 1	0.931-2.396 0.607-1.357 0.718-1.743 0.537-1.565 0.814-1.750 0.764-1.905 0.478-1.451 0.832-2.427 0.442-1.298 0.610-1.453 0.009-0.127 0.024-0.110 0.042-0.157	0.096 0.637 0.620 0.750 0.366 0.421 0.518 0.198 0.312 0.787 0.000 0.000 0.000	1.157 1 0.768 1 1.972 1 0.817 1 1.503 1 2.553 1 1.298 1 2.357 1 1.012 0.983 1 0.180 0.272 0.397 1	0.686-1.953 0.489-1.207 1.200-3.239 0.459-1.454 0.969-2.329 1.527-4.268 0.720-2.339 1.304-4.261 0.542-1.888 0.606-1.594 0.041-0.787 0.133-0.558 0.224-0.705	0.585 0.253 0.007 0.492 0.069 0.000 0.386 0.005 0.971 0.943 0.023 0.000 0.002

CI, confidence interval; CR, complete response; HAI, hepatic arterial infusion; OS, overall survival; PD, progressive disease; PFS, progression-free survival; PR, partial response; SD, stable disease.

### Safety

Treatment-related adverse events were evaluated in all patients (**Table 4**). The most common haematological adverse events were anaemia (34%), leucopenia (33%), and thrombocytopenia (40%) in both arms. The incidence of grade 3 or 4 neutropenia was 2% and 3% in the FOLFOX and TOMOX arms, respectively. Febrile neutropenia was not noted. Elevation of liver enzymes (including alanine amino transferase and aspartic acid amino transferase) was the most frequent non-haematological adverse event, which was seen in 87% and 100% of patients in the FOLFOX and TOMOX arms, respectively; occurrence of grade 3 or 4 elevation of liver enzymes did not significantly differ between the two arms (12% and 18%, respectively; P = 0.432). Bilirubin elevation was seen in 71% and 64% of the patients in the FOLFOX and TOMOX arms, respectively. Grade 3 or 4 hyperbilirubinemia occurred in 4% and 8% of the patients in the FOLFOX and TOMOX arms, respectively, but none of them required biliary stents to relieve jaundice. Approximately half of the patients in both arms experienced severe abdominal pain during agent infusion. Opioid oral administration or lidocaine pumped through the hepatic artery could significantly relieve pain. The incidence of other common clinical adverse events such as nausea, vomiting, fatigue, fever, and diarrhoea was similar in both arms. There were no treatment-related deaths in both arms.

**Table 4:** Summary of safety data.

Adverse event, n (%)	TOMOX (n=61)		FOLFOX (n=52)		P-value^a^
	All Grade	Grade 3-4	All grade	Grade 3-4	
**Hematological**					
Anaemia	21 (34)	0 (0)	13 (34)	0 (0)	0.290^c^
Leucopenia	17 (28)	3 (5)	16 (31)	1 (2)	0.623^b^
Neutropenia	5 (8)	2 (3)	7 (13)	1 (2)	1.000^b^
Thrombocytopenia	20 (33)	4 (7)	20 (38)	1 (2)	0.372^b^
**Nonhematological**					
Elevation of liver enzymes	50 (82)	11 (18)	39 (75)	6 (12)	0.432^b^
Elevation of bilirubin	34 (56)	5 (8)	35 (67)	2 (4)	0.449^b^
Nausea vomiting	10 (16) 13 (21)	0 (0) 0 (0)	11 (21) 9 (17)	0 (0) 0 (0)	0.629^c^ 0.853^c^
Fatigue (asthenia)	10 (16)	0 (0)	5 (10)	0 (0)	0.442^c^
Abdominal pain	32 (52)	31 (51)	31 (60)	27 (52)	1.000^b^
Fever	28 (46)	0 (0)	20 (38)	0 (0)	0.533^c^
Diarrhea	5 (8)	0 (0)	9 (15)	1 (2)	0.460^b^

a P-value calculated using a χ2 test; ^b^p-value comparing Grade 3-4 adverse events; ^c^p-value comparing all Grade adverse events as no patients experienced Grade 3-4 of these adverse events.

## Discussion

HAI of 5-Fluorouracil or oxaliplatin has been proved a safe and feasible treatment even for heavily pre-treated CRCLM (23, 24). However, there is no standard treatment protocol for HAI. The vast majority of studies revolve around the combination of HAI with systemic chemotherapy or targeted therapy, or HAI versus systemic therapy. No head-to-head randomised controlled studies compared two HAI regimens. We had previously performed a retrospective analysis during May 2013 to April 2015 to compare the efficacy and safety of HAI of TOMOX with FOLFOX for patients with unresectable CRCLM. The retrospective analysis showed that the OS was 15.4 versus 20.6 months (P = 0.734) and that the PFS was 6.6 versus 4.9 months (P = 0.215) for the FOLFOX versus TOMOX arms (20). On the basis of that study, we performed this prospective randomised controlled trial for more in-depth analysis of HAI of TOMOX and FOLFOX.

In the current prospective randomised controlled trial, most of the patients had tumours that were refractory to oxaliplatin- and fluorouracil-based chemotherapy, more than 60% patients had tumours that did not response to irinotecan-based therapy and more than half of the patients had exposure to targeted biologic therapy, including bevacizumab and cetuximab. HAI of TOMOX or FOLFOX led to an ORR of 24% for all the patients. The median PFS was 4.6 and 5.8 months in the TOMOX and FOLFOX arms, and the median OS was 13.1 and 17.6 months. The results were consistent with previous studies at other centres. In a randomised phase-II study of HAI of TOMOX for cases of metastatic colorectal cancer wherein the therapy failed or the patients were intolerant to standard systemic therapy, the OS and the PFS were found to be 11.2 and 6.7 months, respectively (19). In this trial, for the patients received HAI as third-line and above therapy, the median PFS was 5.9 months in the FOLFOX arm and 4.4 months in the TOMOX arm, the median OS was 17.8 months in the FOLFOX arm and 12.9 months in the TOMOX arm. For colorectal tumours that are refractory to all the standard first- and second-line systemic therapies, the third-line treatment options are limited. In a prospective phase II study, raltitrexed combined with S-1 treated metastatic colorectal cancer after the failure of conventional chemotherapy demonstrated favourable effects. The median PFS and median OS 2.5 and 8.0 months, respectively (25). Besides, TAS-102 and regorafenib are the recommended third-line systemic therapy. TAS-102 has been reported improve median OS from 5.3 to 7.1 months and median PFS from 1.7 to 2.0 months (26, 27). CORRECT and CONCUR reported that regorafenib versus placebo improved the OS from 5.0 to 6.4 months and 6.3 to 8.8 months, respectively (28, 29). HAI has a considerable survival benefit as a third-line treatment for CRCLM, but large prospective randomized controlled studies are needed to compare the efficacy of HAI and the current standard third-line system therapy in liver-dominant metastases.

HAI is a local therapy specific to liver lesions; peripheral blood concentrations in chemotherapeutic regimens decrease because of the hepatic first-pass effect. In this trial, 58 (51.3%) patients had extrahepatic metastases. The limitation of this study was that there was no combination of systemic or targeted therapy to control extrahepatic metastases more effectively. For KRAS wild-type colorectal cancer, cetuximab combined with chemotherapy as first-line therapy can significantly improve survival time (30, 31). BRAF mutant-type colorectal cancer does not benefit from cetuximab therapy (32, 33). Continued anti-angiogenesis therapy with bevacizumab beyond initial progressive disease is closely related to improvement in survival time (34, 35). In a rat model of colorectal liver metastasis, locoregional application of oxaliplatin and bevacizumab was found to be more effective in reducing tumour growth than systemic treatment with these two agents (36, 37). A retrospective study has showed that HAI combined with systemic chemotherapy and targeted therapy is effective in CRCLM (38). Base on the RAS and BRAF gene status, HAI combine with appropriate targeted therapy is a feasible way, especially for the CRCLM patient with extrahepatic metastases.

TOMOX significantly decreased the duration of bed rest, thereby reducing thromboembolic events caused by immobilization. For elderly patients or patients at high risk of thrombosis, HAI of TOMOX was found to be a better choice.

## Conclusion

HAI chemotherapy was safe and effective for unresectable CRCLM. HAI of FOLFOX has similar efficacy to TOMOX, and HAI of TOMOX had shorter arterial infusion time.

## Data availability statement

The original contributions presented in the study are included in the article. Further inquiries can be directed to the corresponding author.

## Ethics statement

The studies involving human participants were reviewed and approved by Beijing Cancer Hospital Medical Ethics Committee. The patients/participants provided their written informed consent to participate in this study.

## Author contributions

Contributorship statement: XZ and SG designed the research. A-WF, J-HG, F-XK, S-XL, PL, H-FX, and GC collected the data. A-WF analyzed the data and wrote the original draft. J-HG edited language and proofread this manuscript. XZ, X-DW, and HC critically revised the manuscript for important intellectual content. A-WF and J-HG contributed equally to this work and should be considered co-first authors.

## Funding

National Natural Science Foundation of China (81971717).

## Conflict of interest

The authors declare that the research was conducted in the absence of any commercial or financial relationships that could be construed as a potential conflict of interest.

## Publisher’s note

All claims expressed in this article are solely those of the authors and do not necessarily represent those of their affiliated organizations, or those of the publisher, the editors and the reviewers. Any product that may be evaluated in this article, or claim that may be made by its manufacturer, is not guaranteed or endorsed by the publisher.
